# The Role of Fine-Needle Aspiration Cytology in the Diagnosis of Basal Cell Carcinoma

**DOI:** 10.5402/2012/132196

**Published:** 2012-03-26

**Authors:** Masoom Kassi, Pashtoon Murtaza Kasi, Abaseen Khan Afghan, Shah Mohammad Marri, Mahwash Kassi, Iqbal Tareen

**Affiliations:** ^1^Department of Pathology, Bolan Medical College, 8-13/36 Kassi Road, Quetta 87300, Pakistan; ^2^Department of Dermatology, Bolan Medical College, Quetta 87300, Pakistan

## Abstract

*Background/Aims*. Basal cell carcinoma (BCC) is the most common malignant tumor of the skin in humans. The diagnosis of BCC is made clinically, which can then be confirmed microscopically. Biopsy or surgical excision of the lesion provides the specimen for histopathological examination, which is the mainstay for diagnosis. Fine-needle aspiration cytology (FNAC) on the other hand is an even simpler procedure, which can provide accurate diagnosis to confirm or exclude the malignancy. *Methods*. Here, we present our experience on the role of FNAC in diagnosing BCC. We were able to recruit 37 patients, of which 35 had BCC. Both FNAC and biopsy were obtained and then interpreted independently of one another. *Results*. Cytology correlated with histopathology in all cases except for 2 in which the yield was deemed inadequate. The sensitivity and specificity of fine-needle aspiration cytology for basal cell carcinoma were 94.3% and 100%, respectively. *Conclusions*. We, therefore, recommend this technique for the initial evaluation of a patient with suspected BCC or in cases of recurrence. The technique is cheap, quick, less invasive, and highly accurate for the diagnosis of BCC. The limitation of the technique is low yield in some of the cases.

## 1. Introduction

Basal cell carcinoma (BCC) is the most common malignant tumor of the skin in humans. Even though the neoplasm is malignant, it rarely metastasizes, presenting mainly with a healing and recurring lesion, which may bleed as well [1]. The tumor mainly presents in individuals older than 40 years, with the incidence being more in males than females. One of the factors is prolonged “heavy” exposure to sun during youth predisposing to BCC later in life [2]. The diagnosis of BCC is made clinically, which can then be confirmed microscopically [2]. “Suspicious lesions occurring in high risk areas, such as the central portion of the face, should undergo prompt biopsy to obtain a timely diagnosis” [3]. 

Biopsy or surgical excision of the lesion provides the specimen for histopathological examination, which is the mainstay for diagnosis. Fine-needle aspiration cytology on the other hand is an even simpler procedure, which can provide accurate diagnosis to confirm or exclude the malignancy. There are, however, very few reports on the utility of this technique for diagnosing BCC. Here, we present our experience on the role of FNAC in diagnosing BCC.

## 2. Methods

Patients with a clinical suspicion of BCC were referred by a dermatologist for microscopic confirmation. The purpose of the study was explained to the patients and informed consent was obtained. Both FNAC and biopsy were obtained and then interpreted independently of one another. The method for FNAC was adopted from Daskalopoulou and colleagues [4]. 24-gauge needles were used for the fine-needle aspirations. Withdrawal of needle alone, without suction, produced sufficient material for cytological examination. Histopathologic specimens were obtained using 4 mm punch biopsy. They were then fixed in 10% formaldehyde, processed, and embedded in paraffin. The staining used for both the examinations was hematoxylin and eosin (H&E).

## 3. Results

### 3.1. Basic Sociodemographic Characteristics

We were able to recruit 37 patients with suspected BCC. There were 26 (70%) males and 11 (30%) females. The mean age of the patients was 44.0 years (standard deviation—6.03 years). 34 (97.1%) of the cases presented as single lesions; only 1 patient (2.9%) presented with multiple lesions. Majority of the lesions 32 (91.4%) were present on the face, while 3 (8.5%) were present on the neck.

### 3.2. Histological Types

35 cases were identified as BCC; 2 were identified as SCC. Cytology correlated with histopathology in all cases except for 2 in which the yield was deemed inadequate and the result inconclusive. These were shown to be BCC by histopathological examination. Of the five different histological subtypes of BCC, the three identified in our series were nodular 17 (49%), superficial 11 (31%), and micronodular 7 (20%).

### 3.3. Characteristics Seen

Features suggestive of BCC included increased numbers of small, oval cells with hyperchromatic nuclei. The cell clusters had a very thin rim of cytoplasm with a high nuclear to cytoplasm ratio [5]. Some also showed peripheral palisading.

### 3.4. Sensitivity and Specificity of FNAC

Taking histopathology as the gold standard, the sensitivity and specificity of fine-needle aspiration cytology for basal cell carcinoma were 94.3% and 100%, respectively. The positive and negative predictive values were 100% and 50%, respectively.

## 4. Conclusions

Basal cell carcinomas are the most common type of skin cancer, making up more than 80% of the nonmelanoma cancers. The most common location for the tumor, as in our study as well, is the head and the neck.

In our study, FNAC was the investigation under study. It showed both a high sensitivity and specificity in the diagnosis of malignant skin tumors, specifically BCC [6]. We, therefore, recommend this technique for the *initial *evaluation of a patient with suspected BCC or in cases of recurrence. The technique is cheap, quick, less invasive, and highly accurate for the diagnosis of BCC. However, “cytology does not give much information about tumor patterns or subtypes which can be related to aggressive behavior and can be very important in further therapeutic decisions” [6]. This should, thus, be followed by “histopathological confirmation before any therapeutic maneuver is considered” [6]. 

The limitation of the technique is low yield in some of the cases (5.7%). Although most of the patients seen in our series were of the nodular subtype, the technique of FNAC was easier to perform in this subset of patients.

## References

[1] Habif TP (2004). *Clinical Dermatology: A Color Guide to Diagnosis and Therapy*.

[2] Wolff K, Johnson RA, Suurmond D (2005). *Fitzpatrick's Color Atlas & Synopsis of Clinical Dermatology*.

[3] Rubin AI, Chen EH, Ratner D (2005). Current concepts: basal-cell carcinoma. *The New England Journal of Medicine*.

[4] Daskalopoulou D, Maounis N, Kokalis G, Liodandonaki P, Belezini E, Markidou S (1993). The role of fine needle aspiration cytology in the diagnosis of primary skin tumors.. *Archives d’Anatomie et de Cytologie Pathologiques*.

[5] Fang X, Ma B (1999). Fine needle aspiration cytology of basal cell carcinoma of the skin: a clinical and cytopathological appraisal. *The Journal of Dermatology*.

[6] Vega-Memije E, Martinez-De Larios N, Waxtein LM, Dominguez-Soto L (2000). Cytodiagnosis of cutaneous basal and squamous cell carcinoma. *International Journal of Dermatology*.

